# Experimental Therapies in Multiple Sclerosis: Epstein–Barr Virus and Potential EBV-Related Therapeutic Strategies—A Systematic Review

**DOI:** 10.3390/jcm15114104

**Published:** 2026-05-26

**Authors:** Julia Bartczak, Piotr Gronowski, Martyna Małek, Aleksandra Denkiewicz, Olga Grodzka, Piotr Chądzyński, Izabela Domitrz

**Affiliations:** 1 Department of Neurology, Faculty of Medicine and Dentistry, Medical University of Warsaw, 01-809 Warszawa, Poland; 2 Doctoral School, Medical University of Warsaw, 02-091 Warszawa, Poland

**Keywords:** EBV, EBV-transformed cells, Epstein–Barr virus, multiple sclerosis, MS, experimental therapy, immunotherapy, targeted therapy

## Abstract

**Background/Objectives:** Multiple sclerosis (MS) constitutes a chronic autoimmune, inflammatory, and neurodegenerative disease, with dissemination in space and time, warranting diagnosis. Epstein–Barr virus (EBV) is increasingly recognized as a key contributor to MS pathogenesis. This review summarizes evidence on EBV-related mechanisms of currently approved disease-modifying therapies (DMTs) and emerging EBV-directed therapeutic strategies in MS. **Methods:** A systematic search of PubMed, Embase, Cochrane, and Web of Science was performed. Original English-language studies addressing EBV-related therapeutic mechanisms or EBV-targeted interventions in MS were included; 23 studies met the inclusion criteria. **Results:** Current DMTs may influence EBV-related immunity through diverse mechanisms, including modulation of B-cell subsets, altered lymphocyte trafficking, reduction in EBV-specific humoral responses, and restoration of T-cell surveillance. Monoclonal antibody-based therapies, particularly anti-CD20 agents and natalizumab, appear to affect the EBV–B-cell–immune axis through distinct but complementary mechanisms. Other interventions, including interferons, glatiramer acetate, dimethyl fumarate, autologous hematopoietic stem cell transplantation, and vitamin D supplementation, may also modulate EBV-specific cellular or humoral responses, although the magnitude and durability of these effects vary. Emerging EBV-directed approaches, including EBV-specific T-cell therapy, inhibition of specific proteins, modulation of autophagy, and cholesterol-dependent viral latency, provide additional support for targeting EBV-related pathways in MS. **Conclusions:** The therapeutic efficacy of DMTs in MS may extend beyond nonspecific immunomodulation and involve partial disruption of EBV-driven immune persistence. Further controlled studies are required to validate EBV-related biomarkers and determine whether direct EBV-targeted therapies can provide sustained clinical benefit.

## 1. Introduction

### 1.1. Multiple Sclerosis: Immunopathogenesis and Limitations of Current Disease-Modifying Therapies

Multiple sclerosis (MS) is a chronic autoimmune, inflammatory, and neurodegenerative disease of the central nervous system (CNS) that affects over 2.8 million people worldwide [1,2]. The diagnosis of MS is based on the demonstration of dissemination of lesions in space and time within the CNS, while excluding alternative diagnoses [3]. The most recent revision of the McDonald criteria [3] further refines the role of magnetic resonance imaging (MRI) and paraclinical findings, including cerebral vein sign and paramagnetic rim lesions in MRI or cerebrospinal fluid-specific (CSF) oligoclonal bands. Importantly, in selected clinical scenarios incorporating these MRI biomarkers and paraclinical findings, the updated criteria allow for MS diagnosis without mandatory demonstration of dissemination in time, thereby facilitating earlier initiation of disease-modifying therapy (DMT) while maintaining diagnostic specificity [3]. MS remains the leading cause of non-traumatic neurological disability among young adults [2,4]. The pathogenesis of MS is driven by multifactorial interaction between genetic susceptibility and environmental triggers, which collectively prime autoimmune responses against CNS antigens [1,2,4,5].

From an immunopathological point of view, MS lesions are characterized by infiltration of autoreactive T lymphocytes (both CD4^+^ and CD8^+^), B-cells, and plasma cells, alongside activated cells of the innate immune system, leading to demyelination, axonal loss, and gliosis [5,6,7]. In addition to their humoral role, B-cells significantly contribute to disease pathogenesis through antigen presentation, cytokine signaling, and the formation of ectopic lymphoid-like structures within the meninges that may sustain intrathecal inflammation. [6,7,8]. Neurodegeneration and clinical progression are increasingly linked to compartmentalized processes within the CNS, including mitochondrial dysfunction, oxidative stress, and microglial activation [1,2,4,5,6,7].

Over recent decades, the introduction of DMTs has transformed the management of MS, primarily its relapsing–remitting type (RRMS); however, in the last few years, DMTs for other MS types have also been more commonly used. These agents effectively suppress clinical relapses and radiological activity while mitigating the risk of long-term disability progression [9]. Despite these advances, significant challenges remain. The efficacy of current immunomodulators, which primarily target peripheral pathways, is notably reduced as the disease progresses and inflammation becomes compartmentalized within the CNS, coinciding with ongoing neurodegenerative processes [7,9,10]. Current treatments rarely provide complete disease control and lack the capacity to reverse established tissue damage. Their long-term utility is frequently limited by significant adverse effects [9,11]. Identifying novel therapies that target CNS-specific pathways to complement systemic immunomodulation remains a clinical priority.

### 1.2. Role of Epstein–Barr Virus in Multiple Sclerosis

Epstein–Barr virus (EBV) is a prevalent human lymphotropic herpesvirus with a well-recognized causal role in several types of cancer [12,13,14,15]. EBV establishes a persistent latent infection in B lymphocytes through a combination of viral reprogramming of B lymphocytes and encoding a wide array of immune response-modulating proteins [16]. Prospective studies using blood samples collected several years before MS onset showed that higher immunoglobulin G (IgG) titers against viral capsid antigens (VCA) and EBV nuclear antigens (EBNA) were associated with a significantly increased risk of developing MS. Individuals with the highest EBV antibody levels had a markedly higher risk of MS compared with those with the lowest levels [17].

EBV-specific T-cell and antibody responses, particularly those targeting EBNA1, demonstrate cross-reactivity with host CNS antigens. This molecular mimicry initiates a neuroinflammatory response, in which the immune system accidentally attacks myelin or neuronal structures while attempting to eradicate viral antigens [18]. Furthermore, EBV infection significantly reshapes lymphocyte dynamics. Virus alters B-cell maturation, promoting the survival of autoreactive B-cell clones and enhancing chronic pro-inflammatory interactions between B- and T-cells [19]. Latent EBV infection persists in memory B-cells, which can infiltrate the CNS and form ectopic follicle-like structures in the meninges [19,20]. These reservoirs promote local neuroinflammation by releasing viral products, such as ribonucleic acid (RNA) and inflammatory exosomes, which maintain a state of chronic immune activation [6].

The pathogenesis is intensified by genetic risk factors, most notably the major histocompatibility complex (MHC) class II haplotype human leukocyte antigen (HLA)-DRB1∗15:01. This risk allele contributes to higher viral load and modulates anti-EBV immune responses. The synergy between this genetic predisposition and symptomatic EBV infection significantly elevates the risk of MS development [21]. Collectively, the current research points to EBV as a major contributor to MS pathogenesis, providing a framework for understanding disease onset and progression.

### 1.3. Rationale for Targeting EBV-Transformed Cells

As described above, there is much evidence supporting the role of EBV infection in the pathogenesis of MS [18,19,20,21,22]. Therefore, it is suggested that targeting EBV-infected cells may represent a potential therapeutic strategy.

Various methods are suggested to positively impact the course of MS by influencing EBV-related mechanisms. Some of the therapeutic strategies have focused on vaccines designed to induce EBV-specific immune responses and limit viral persistence [23]. For instance, in one promising study, a vaccine targeting the viral surface glycoprotein 350 (gp350), which allows the virus to enter B-cells through binding to the CD21 receptor, was introduced [24,25]. In a phase II clinical trial, this gp350-based vaccine reduced the incidence of infectious mononucleosis by approximately 78%, although prevention of asymptomatic EBV infection was not completely achieved [25]. These therapies may prevent primary infection or reduce its severity and decrease the risk of developing MS [24]. Another example of a therapeutic strategy to inhibit EBV replication is using other antiviral drugs such as acyclovir, ganciclovir, and tenofovir. These drugs do not affect latently infected cells, but they block virus replication, limit production of new virions, and reduce the infection of new cells [26].

In the treatment of MS, there are effective immunosuppressive and anti-inflammatory therapies that prove autoimmune mechanisms in disease pathogenesis [16]. Increasing evidence indicates that B-cells play a central role in MS, as it has already been demonstrated by the clinical success of B-cell-depleting therapies targeting CD20, such as ocrelizumab and ofatumumab [27]. Early clinical studies demonstrated that EBV-specific T-cell therapy may lead to clinical improvement in some patients with progressive MS [16].

### 1.4. Aim of the Study

Given the above, this systematic review aims to illustrate the important role of EBV in the pathogenesis of MS and discuss new experimental treatment strategies targeting EBV-infected and EBV-transformed cells. Furthermore, the EBV influence on the mechanisms of action of already approved therapies has been explored. Available experimental and clinical evidence on EBV-directed therapies has been summarized, highlighting their potential as innovative therapeutic strategies for the treatment of MS.

## 2. Methodology

This review was written systematically in accordance with the Preferred Reported Items for Systematic Reviews and Meta-Analyses (PRISMA 2020) guidelines [28]. The PRISMA 2020 checklist can be found in Supplementary Table S1. This study’s protocol was registered in PROSPERO, an international prospective register of systematic reviews, on the 2nd of May 2026 (registration ID: CRD420261385274).

### 2.1. Inclusion and Exclusion Criteria

Specific inclusion and exclusion criteria have been applied. To be included, an article had to address EBV-related therapy in patients with MS. Studies that did not include patients with MS or treatment interventions involving EBV were not allowed. Original studies of any type were included; thus, studies providing the analysis and synthesis of already published studies, such as reviews (narrative or systematic) or meta-analysis, as well as other article types (commentaries, guidelines) that did not present original data regarding EBV-related treatment in MS, were excluded. Additionally, case reports were not allowed. We included studies written in English to ensure that the broad readership can understand the cited sources; therefore, studies in languages other than English met the exclusion criteria. In case of any uncertainties, a discussion involving all authors would be performed to resolve them; however, no such situation appeared.

### 2.2. Selection Process

**Figure 1:**
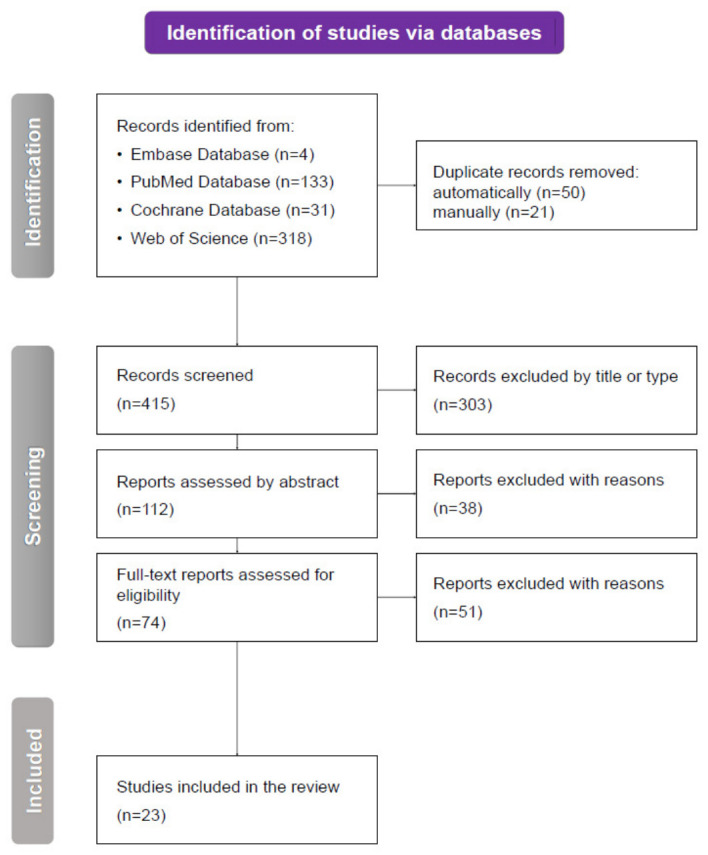
A flowchart presenting the selection process graphically. n, number.

The selection process was conducted independently by two authors (J.B. and P.G.), and any discrepancies between the authors were resolved by the third author (O.G.). EndNote 21 (Clarivate, Philadelphia, PA, USA) was used for the selection process, duplicate removal, and reference management. To find the most adequate studies, four databases were searched, which were as follows: PubMed Database, Embase Database, Cochrane Database, and Web of Science Database, with the search strategy, as follows: (‘experimental treatment’ OR ‘experimental therapy’ OR ‘treatment trial’) AND (‘multiple sclerosis’ OR ‘MS’ OR ‘sclerosis multiplex’) AND (‘EBV’ OR ‘epstein-barr virus’ OR ‘human herpesvirus 4’ OR ‘HHV-4’). This search led to the identification of 485 potentially related articles, 133, 4, 31, and 318 from each database, respectively. After the automatic removal of 50 duplicates, the other 435 records were screened. Thus, 303 articles were removed by title or type, 38 by abstract, and 48 by the full text. Furthermore, for three studies, although the abstract was in English, the full text was written in another language. Throughout the search, 21 additional duplicates were manually assessed as duplicates. Finally, 23 studies were included in the following review. The full selection process is presented in Figure 1.

### 2.3. Data Curation and Data Synthesis

Primary data curation was performed by two authors (J.B. and P.G.), followed by any discrepancy resolution by the third author (O.G.). All studies were sought for general data, such as patient population, comparison, and EBV-related treatment method type. In case of research analyzing EBV-based therapies, the data indicated from the studies included baseline disease severity, improvement after applied treatment (which was measured by clinical and neurological examination, the Expanded Disability Status Scale (EDSS), assessment of MRI, and/or CSF results), and time to improvement. In cases when follow-up was performed, the time of follow-up and the change in outcomes over time were noted. In situations when the research addresses specific EBV-related mechanisms of already known MS therapies, studies were sought for information, including changes and alterations in specific protein levels, and their impact on the studied population.

The systematic curation of the collected data enabled the authors to divide the review into appropriate subsections and analyze works addressing specific subtopics together. Furthermore, although several systematic methods have been applied, data synthesis has been performed primarily narratively. The significant heterogeneity among studies, including study design, population characteristics, assessment methods, and, most crucially, EBV-related therapy type, was noted; therefore, a narrative synthesis was the most suitable method for between-studies comparisons.

### 2.4. Risk of Bias Assessment

To assess the risk of bias in the included studies, the Newcastle–Ottawa Scale was used. Each study was assessed primarily by one of the authors (J.B., P.G., M.M., or A.D.) and later verified by the other authors to achieve overall agreement. In case of discrepancies, the assessment was verified by the other author (O.G.). All of the studies were assessed as low (7–8 points out of 9) or moderate (4–6 points) risk of bias; however, caution remains warranted when concluding, especially when analyzing studies rated with the lowest scores (4 or 5 out of 9). The complete assessment of the risk of bias for each study can be found in Supplementary Table S2.

## 3. Results

### 3.1. The Role of EBV in the Mechanisms of Action of MS Therapies

The clinical success of DMTs in multiple sclerosis (MS) has been attributed to their broad immunomodulatory and anti-inflammatory effects. Given the increasingly indisputable etiological link between Epstein–Barr virus and MS pathology, it is essential to evaluate the degree to which the efficacy of these established therapies depends on their impact on the EBV–host dynamic. Recent evidence suggests that the therapeutic benefit of several DMTs may be partially mediated through a mechanistic synergy, simultaneously dampening neuroinflammation and re-establishing control over latent viral infection. By targeting the pathogenic B-cell reservoir, suppressing EBV-specific hyper-responsiveness, or reinvigorating exhausted cytotoxic surveillance, these therapies potentially disrupt the EBV-driven inflammatory cascade at its source. This section explores how conventional treatments, ranging from early first-line injectables to intensive immune-reconstitution protocols, interact with the viral life cycle and the specialized immune cells that govern its latency.

#### 3.1.1. Monoclonal Antibodies

Natalizumab is an integrin antagonist that inhibits lymphocyte trafficking in patients with RRMS [28]. It has been investigated in three mechanistic studies for its indirect modulation of EBV-associated immune responses in MS [29,30,31]. Across studies in RRMS, natalizumab-treated patients showed consistent changes in peripheral B-cell distribution, characterized by increased frequencies of circulating memory B-cells and antibody-secreting cells, reflecting reduced lymphocyte trafficking across the blood–brain barrier [29,30,31]. Ex vivo stimulation of peripheral blood mononuclear cells (PBMCs) with interleukin 2 (IL-2) and R848 presented augmented Ig production in natalizumab-treated patients, including increased total IgG and IgM secretion compared with untreated RRMS and healthy donors [31]. Importantly, Ig secretion per single antibody-secreting cell did not differ significantly between groups, indicating, in particular, that natalizumab affects, predominantly, the number of antibody-producing cells rather than their per-cell functional capacity [29,31].

One of the mentioned studies presented that natalizumab therapy was linked to an increased EBV-specific humoral response, indicated by higher IgG reactivity against EBNA1. This effect was most evident for antibodies targeting the C-terminal region of EBNA1 (aa380–641), which had previously been associated with molecular mimicry involving MS-associated autoantigens. Increased EBNA1-directed responses were linked to increased reactivity against candidate autoantigens such as anoctamin 2 and glial cell adhesion molecule (GlialCAM), suggesting a potential connection between EBV-specific antibodies and CNS autoimmunity [31]. Another research group found that beyond antigen-specific findings, natalizumab treatment was associated with a redistribution of the peripheral B-cell compartment, characterized by increased circulating memory B-cells and antibody-secreting cells, which is consistent with impaired lymphocyte migration into the CNS [29]. In another study, natalizumab treatment showed altered peripheral immune cell dynamics, with accumulation of activated B-cell subsets in circulation and elevated humoral immune responsiveness after in vitro stimulation [30]. Collectively, although natalizumab does not directly target EBV, mechanistic evidence suggests that it modulates EBV-directed humoral immunity indirectly through altered immune cell trafficking and peripheral retention of memory B-cells, enhancing detectable EBV-specific antibody responses in the periphery [29,30,31].

Ocrelizumab and rituximab, as anti-CD20 monoclonal antibodies, target B-cells, leading to B-cell depletion and functional modulation. They are increasingly investigated in the context of their potential impact on EBV infection [32,33]. In the first of these studies, ocrelizumab treatment was associated with a selective reduction in humoral EBV responses, including decreased antibodies against EBNA-1 and BamHI F rightward reading frame 3 protein, without changes in those against gp350 or total IgG levels [33]. It suggests a selective modulation of the anti-EBV response despite changes in the B-cell compartment. A decreasing trend was also observed for antibodies against selected EBV cross-reactive human proteins (septin-9, dihydrolipoamide S-succinyltransferase). However, heterogeneous nuclear ribonucleoprotein L remained unchanged, suggesting that not all cross-reactive autoantigens are affected equally [33]. In a prospective cohort of 99 patients treated with ocrelizumab or rituximab, EBNA1 IgG decreased by approximately 12–15% over 6–18 months, while viral capsid antigen IgG increased by approximately 12–14% starting from month 3 [32]. Additionally, cytomegalovirus (CMV) IgG and total IgG decreased moderately, suggesting a partial nonspecific reduction in Ig levels, and EBV deoxyribonucleic acid (DNA) was rarely detected (3/156 samples). Higher initial EBNA1 IgG levels were observed in HLA-DRB1*15:01-positive patients [32]. Overall, anti-CD20 therapy, both ocrelizumab and rituximab, selectively affects the humoral immune response to EBV, which may reflect a reduction in the EBV reservoir within B-cells and a weakening of chronic antigenic stimulation, potentially limiting molecular mimicry mechanisms and the persistence of autoimmune processes in MS [32,33].

Another monoclonal antibody, daclizumab high-yield process (DAC HYP), although not developed as an antiviral agent, has been investigated as an immunomodulatory therapy in RRMS [34]. DAC HYP treatment was associated with a significant normalization of compartment-specific T-cell immunity. It reduced EBV-specific CD4+ and CD8+ T-cell responses in the CSF and increased EBV-specific T-cell responses in peripheral blood. This shift was accompanied by a pro-inflammatory cytokine production decrease (mostly interferon gamma (IFN-γ) and tumor necrosis factor alpha (TNF-α)) within the CSF compartment [34]. These findings indicate that DAC HYP may indirectly modulate EBV-driven immunopathology in MS by altering lymphocyte trafficking rather than directly targeting the virus itself. It suggests that EBV-specific immune responses are involved in the inflammatory processes underlying MS.

Finally, Jagessar et al. examined how different B-cell-targeting therapies affect disease development in a marmoset model of MS, an experimental autoimmune encephalitis [35]. The authors focused on virus-infected B-cells. Monkeys were treated with anti-CD20 and with antibodies against B-lymphocyte stimulator (BLyS) and a proliferation-inducing ligand (APRIL), while untreated animals were a control group. The study also assessed the presence of a γ-herpesvirus, CalHV3, which is an animal virus similar to EBV. Anti-CD20 treatment significantly reduced the number of virus-infected B-cells and was associated with clinical improvement, whereas anti-BLyS and anti-APRIL therapies were less effective and did not reduce viral load. Additional experiments showed that virus-infected B-cells can activate autoreactive T-cells and promote inflammation in the CNS. Overall, virus-infected B-cells may play an important role in disease development and could be a key target of effective therapies [35]. Table 1 summarizes all studies cited in Section 3.1.1 with some additional information.

#### 3.1.2. Other Disease-Modifying Therapies

IFN-β, a long-standing first-line therapy for RRMS, possesses well-known antiviral effects. In the context of EBV, its mechanism appears to be centered on the depletion of the viral reservoir, as presented in various studies. Rizzo et al. demonstrated that IFN-β therapy specifically induces apoptosis in CD27+ memory B-cells, the primary site of EBV latency, via a FAS-mediated pathway [36]. This selective reduction in the memory B-cell compartment correlates with a significant decrease in the expression of the EBV latent gene, precisely latent membrane protein 2A. This protein acts as a functional mimic of the B-cell receptor signaling, providing infected B-cells with essential survival signals even in the absence of antigen; therefore, its downregulation by IFN-β effectively deprives the virus of its mechanism for long-term persistence within the host [36]. Furthermore, IFN-β also modulates the activity of plasmacytoid dendritic cells (pDCs), which are found in high concentrations within MS brain lesions in proximity to EBV-infected cells [37]. These pDCs serve as a local source of type I IFNs, as evidenced by the abundant expression of the IFN-induced protein myxovirus resistance protein 1 in their vicinity. Chronic IFN-β therapy appears to impair the maturation of these pDCs and regulates their responses mediated by Toll-like receptors, potentially shifting the local CNS microenvironment toward a more anti-inflammatory state and limiting the stimulus for chronic B-cell activation [37]. Despite these profound cellular and molecular effects, longitudinal studies indicate that IFN-β treatment does not significantly alter the humoral response to EBV. The recent analysis of the OFAMS study cohort demonstrates that 18 months of IFNβ-1a treatment showed no significant associations with serum levels of EBNA-1 IgG, EA IgG, and VCA IgG or VCA IgM [38]. This follow-up period is considered sufficient to capture potential treatment-mediated effects on the humoral response, a notable distinction from other disease-modifying therapies like ocrelizumab or teriflunomide, which have been shown to attenuate these antibody titers [38]. Moreover, while the HLA-DRB1*15 risk allele is associated with higher mean EBNA-1 IgG levels, it does not influence the stability of these antibody titers during interferon therapy according to the study by Lie et al. [39]. Serum levels of IgG antibodies against EBNA-1, EA, and VCA remain remarkably stable during treatment, reinforcing the suggestion that the therapy’s impact is confined to the cellular reservoir and innate immune sensors rather than the established humoral memory [39].

The immunomodulatory mechanism of other medication, glatiramer acetate (GA), has traditionally been associated with a Th1-to-Th2 phenotype shift. Research by Guerrera et al. indicates that GA plays a crucial role in restoring immunological surveillance against EBV [40]. Specifically, GA therapy has been shown to increase the frequency of circulating EBV-specific CD8+ T lymphocytes, which are essential for controlling the expansion of EBV-infected B-cells. Importantly, this effect goes beyond a simple quantitative increase; GA treatment actively reverses the “immune exhaustion” typically observed in MS patients. Simultaneously, GA therapy exerts a profound influence on the B-cell compartment, promoting a shift from CD27+ memory B-cells, the primary reservoir for EBV latency, toward a more naïve B-cell population. This dual action creates a synergistic therapeutic effect: while the “predatory” CD8+ T-cell response is strengthened and its exhaustion reversed, the “prey” (the pool of EBV-infected memory B-cells) is significantly reduced. This mechanistic interplay likely contributes to the reduction in EBV-driven inflammatory triggers and the stabilization of the disease course in patients treated with GA [40].

Dimethyl fumarate (DMF) is known for its antioxidant and anti-inflammatory properties, but its impact on EBV-specific cellular immunity is particularly significant. In the previously mentioned study by Dungan et al., the authors reported that DMF treatment was associated with a significant suppression of the cellular immune response directed against EBNA-1 peptides [30]. Patients treated with DMF exhibit noticeably reduced production of pro-inflammatory cytokines when exposed to EBNA-1 antigens. This finding is of profound clinical significance as it suggests that DMF “normalizes” the pathologically exaggerated T-cell reactivity toward EBV. This immunomodulatory effect exhibits a high degree of antigen specificity. While the T-cell response to EBNA-1 is suppressed, the cellular response to other ubiquitous herpesviruses, such as CMV, remains largely unaffected. This indicates that DMF does not induce generalized immunosuppression but rather targets the specific T-cell clones or pathways involved in the EBV-driven inflammatory cascade. Furthermore, mirroring the patterns observed with IFN-β, the humoral response (anti-EBNA-1 and anti-VCA IgG titers) remains stable during DMF therapy. This dissociation between cellular suppression and humoral stability reinforces the hypothesis that the drug’s interaction with EBV is primarily mediated by the cellular arm of the immune system, specifically by dampening T-cell-mediated hyper-responsiveness to viral antigens without compromising the established B-cell-derived antibody memory [30].

#### 3.1.3. Autologous Hematopoietic Stem Cell Transplantation

In the previously mentioned study by Marti et al., the impact of autologous hematopoietic stem cell transplantation (aHSCT) on the EBV-specific B-cell repertoire was also investigated [31]. As an intensive “immune reset,” aHSCT is designed to eradicate the pathogenic immune system and rebuild it from hematopoietic stem cells. The procedure leads to a profound depletion of the memory B-cell compartment, theoretically eliminating the bulk of the EBV reservoir [31]. However, research by Massey et al. highlights a critical nuance to this process: while the patient’s endogenous B-cells are depleted, the infused leukapheresis graft itself often contains latently EBV-infected memory B-cells [41]. This reintroduction of the virus into an immunocompromised recipient can lead to transient EBV viremia in the first months following the transplant [41].

Despite this viral presence, the already mentioned study by Marti et al. revealed that EBNA-1-specific memory B-cells can still be detected in the reconstituted repertoire following transplantation. This is of significant concern given the molecular mimicry between the EBNA-1 protein and CNS self-antigens, specifically GlialCAM, a protein expressed by oligodendrocytes and astrocytes. The structural similarity between the EBNA-1 epitope and GlialCAM allows cross-reactive B-cells to misidentify CNS tissue as viral targets, thereby potentially perpetuating neuroinflammation [31].

The therapeutic success of aHSCT may therefore rely on a fundamental shift in the cellular immune response to these persistent viral antigens. Massey et al. demonstrated that between 6 and 24 months post-transplant, there is a significant expansion and diversification of the CD8+ cytotoxic T lymphocyte repertoire reactive with both lytic and latent EBV antigens [41]. This robust diversification suggests that aHSCT enables recruitment of new, potentially more effective T-cell clones to monitor the viral reservoir. The authors observed that while the level of latent EBV infection within the B-cell pool may actually increase following treatment (due to the re-infusion and early reactivation), this does not necessarily correlate with disease relapse [41]. This finding supports the hypothesis that the “reset” provided by aHSCT acts not necessarily through total viral eradication, but by restoring a more functional and diverse T-cell-mediated surveillance that can effectively suppress the EBV-driven inflammatory cascade and prevent its cross-reactive attack on CNS proteins like GlialCAM [31,41].

#### 3.1.4. Vitamin D

Vitamin D plays several important roles in the body, one of which is maintaining immune tolerance. Low serum levels of 25-hydroxyvitamin D, its principal metabolite in blood, are considered one of the factors that may increase the risk of developing MS. Vitamin D acts by limiting the proliferation of Th1 CD4+ T-cells and reducing T-cell-dependent antibody production. Dysregulation of these immunomodulatory mechanisms may lead to EBV reactivation in patients with MS [42,43]. Two research groups investigated the effect of vitamin D supplementation on IgG levels against EBNA1 and VCA [42,43]. In the study by Najafipoor et al., patients with RRMS received 50,000 IU of vitamin D per week for six months, while patients with the same diagnosis were included as a control group [42]. Both groups, study and control, were seropositive for anti-EBV antibodies and were receiving interferon therapy. After six months, the study group showed a smaller increase in IgG levels against EBNA1 and VCA compared to the control group [42]. Similarly, thirty-five patients with RRMS received 20,000 IU of vitamin D3 per week for 96 weeks in another study, while thirty-three patients with the same diagnosis received a placebo [43]. Both groups were seropositive for anti-EBV antibodies. The group receiving vitamin D3 showed a decrease in IgG levels against EBNA1 from baseline to week 48 compared with the placebo group; however, this effect was no longer observed at week 96 [43]. These findings suggest that vitamin D supplementation may limit the rise in antibody titers against EBV and, thus, affect (at least temporarily) the immune response against the latent EBV antigen EBNA1 in patients with RRMS [42,43]. All studies described for the first time in Section 3.1.2, Section 3.1.3 and Section 3.1.4, with some additional information, have been summarized in Table 2.

### 3.2. EBV-Based Therapies

#### 3.2.1. EBV-Specific T-Cell Therapy

Expansion of EBV-infected B lymphocytes is considered a potential factor involved in the pathogenesis of MS; therefore, CD8+ T-cell-based therapy targeting EBV-infected cells may represent a promising therapeutic option [44,45]. The first research group evaluated the long-term effects of autologous EBV-specific T-cell therapy in patients with MS, of whom half were diagnosed with primary progressive and the other half with secondary progressive MS [44]. Participants received the therapy and were followed up at 2 and 3 years. Patient evaluation included clinical and neurological examination, EDSS score assessment, MRI, and CSF analysis. The therapy was well-tolerated and associated with sustained clinical improvement in some patients with progressive MS. This effect persisted for up to 2–3 years, but gradually diminished over time, likely due to T-cell depletion and persistent exposure to EBV antigens. The effectiveness of the therapy was associated with the level of T-cell reactivity to EBV [44]. The authors suggested that repeated administration may be necessary to maintain clinical benefit. In another study conducted by Pender et al., autologous latent membrane protein (LMP)/EBNA1-specific T-cells were generated from the blood of patients with progressive MS [45]. The transfer of these lymphocytes was found to be safe and well-tolerated by the study participants. Within 2–14 weeks after the first infusion, six patients showed clinical improvement, including reduced fatigue, enhanced quality of life, and, in some cases, neurological improvement and a decrease in EDSS score [45].

#### 3.2.2. Other EBV-Based Therapies

Among other therapies targeting EBV, EBNA1 inhibitors appear particularly promising. As highlighted in previously discussed studies, EBNA1 is a key nuclear protein responsible for maintaining and replicating the viral genome in B lymphocytes during the latent phase. Monaco et al. investigated the effect of the EBNA1 inhibitor (VK-1727) on PBMCs, particularly EBV+ and EBV− B lymphocytes, and compared its effects with cladribine, a DMT type [46]. PBMCs were collected from patients with MS in stable and active disease and from healthy controls. EBV was more frequently present in CD19+ B lymphocytes in acute MS (approximately 80%) than in the control group (42%) and stable MS patients (28%). The EBNA1 inhibitor suppressed metabolic activity only in EBV+ cells and had no effect on EBV− cells, unlike cladribine, which inhibited both populations non-selectively. VK-1727 predominantly exerted a cytostatic rather than cytotoxic effect, inhibiting the cell cycle in EBV+ cells without inducing significant apoptosis, whereas cladribine strongly induced apoptosis in both EBV+ and EBV− cells. These findings suggest the potential clinical relevance of EBNA1 inhibitors as targeted therapies against EBV-infected cells and support further studies in MS [46]. In the study by Morandi et al., the effect of EBV infection on the processing of the myelin oligodendrocyte glycoprotein (MOG) autoantigen was investigated, a protein relevant to MS pathogenesis [47]. The authors demonstrated that EBV increases the expression of antigen presentation markers HLA I and HLA II (CD80, CD86), which may promote activation of autoreactive T-cells. EBV was shown to induce MOG degradation via Cathepsin G activation and concomitantly induce autophagy, which protects selected MOG epitopes from degradation, promoting their cross-presentation to autoreactive CD8+ T-cells. Therefore, modulation of autophagy and proteolytic enzymes may represent a promising therapeutic strategy in EBV-associated MS [47].

Membrane cholesterol was also demonstrated to play a crucial role in EBV infection of astroglial cells and the maintenance of viral latency [48]. Treatment with methyl-β-cyclodextrin (MbCD) in EBV-infected LN-229 astroglial cells decreased the expression of latent viral genes (LMP1, LMP2A, and EBNA1), indicating impaired maintenance of infection in host cells. Cholesterol depletion by MbCD reduced the expression of signal transducer and activator of transcription 3, receptor-interacting protein, nuclear factor kappa B, and TNF-α proteins, suggesting attenuation of the activation of signaling pathways, utilized by the virus to modulate host cells and induce inflammatory processes. Based on these findings, it may be hypothesized that cholesterol reduction may have potential therapeutic relevance in MS.

In the study by Annunziata et al., clones of EBV-infected B-lymphocytes were derived from patients with MS and healthy individuals to assess the types of antibodies produced within each group. Subsequently, the association between clinical course in MS patients and the presence of anti-myelin basic protein (MBP) monoclonal IgM antibodies and antibodies against other myelin proteins, proteolipid protein, and MOG, was investigated. Furthermore, their epitope specificity and in vitro immunological activity were evaluated. Patients with antibodies against the specifically MBP 105–120 epitope had a longer duration of disease stability, lower relapse rate, and a lower degree of disability. Anti-MBP antibodies 105–120 inhibited T lymphocyte proliferation. In addition, they were shown to bind to the Fc gamma receptor I present on monocytes and macrophages. This interaction increased the production of the anti-inflammatory cytokine IL-10 while decreasing the production of the pro-inflammatory cytokine IL-12. The findings suggest that naturally occurring IgM antibodies with specificity for MBP, exerting immunosuppressive effects, could form the basis of a new therapy for MS. All studies mentioned in Section 3.2, with some additional information, have been summarized in Table 3.

### 3.3. EBV-Related Predictors of Treatment Effectiveness

Currently, there is a strong emphasis on developing models that can predict treatment response. Starting the appropriate therapy early and regularly monitoring disease activity enables timely detection of treatment failure, thus suggesting treatment adjustment. Therefore, identifying prognostic factors and early biomarkers plays a key role in reducing the risk of treatment failure. The following part of the review addresses this issue. Comabella et al., in a prospective study, evaluated EBV-specific humoral and cellular immune responses in patients with RRMS [50]. The study population consisted of twenty-eight patients who responded clinically to IFNβ therapy, defined by the absence of EDSS progression and relapses during the first two years of treatment. IgG responses against EBNA1 and VCA were assessed in 24 patients. IgG levels against EBNA1 showed a non-significant decrease after one year of therapy, while anti-VCA responses remained stable. Cellular analyses from 18 patients demonstrated a marked downregulation of EBNA1-specific CD4+ T-cell responses during IFNβ treatment, whereas CD8+ T-cell responses to other EBV antigens remained unchanged. All immune responses were evaluated at baseline before IFNβ therapy and after 1 year of treatment. These findings indicate that EBNA1-specific CD4+ T-cell responses are reduced early during IFNβ therapy and are associated with the clinical response to IFNβ treatment [50].

Dominguez-Mozo et al. assessed the antiviral effects of teriflunomide in 80 patients with MS by measuring IgG antibody titers against EBNA-1 and VCA at baseline and after 6 months of treatment [51]. They also examined whether changes in these titers were associated with clinical and radiological outcomes after 24 months and searched for early biomarkers of treatment response. IgG titers against EBNA-1 and VCA decreased after 6 months of therapy; however, these changes were not significantly associated with clinical or radiological response at 24 months. However, the authors identified two factors: older age at treatment initiation and higher baseline EBNA-1 titers as potential early predictors of achieving No Evidence of Disease Activity (NEDA-3) status in patients starting teriflunomide [51]. In another study, Dominguez-Mozo et al. sought factors that could predict response to natalizumab in 186 patients with MS [52]. They analyzed clinical, radiological, and immunological variables, including anti-EBNA1 and anti-VCA antibody levels, as well as changes in human herpes virus 6 (HHV-6) IgG titers between baseline and 6 months. Three factors were identified as predictors of early response to treatment, including a baseline EDSS score below 3.0, lower initial levels of anti-EBV antibodies, and changes in HHV-6 IgG titers during the six-month follow-up. In particular, a decrease in HHV-6 IgG levels was associated with a better clinical response [52]. All studies cited in Section 3.3, with some additional information, have been summarized in Table 4.

## 4. Discussion

Up-to-date, available DMTs for MS include interferon beta, glatiramer acetate, teriflunomide, dimethyl fumarate, and sphingosine-1-phosphate receptor modulators such as fingolimod, siponimod, ozanimod, ponesimod, as well as anti-CD20 therapies, including ocrelizumab, ofatumumab, ublituximab, and alemtuzumab targeting CD52 with a broad depletion of T and B lymphocytes, natalizumab targeting alpha4-integrins and cladribine inducing preferential lymphocyte depletion through purine nucleoside analog-mediated apoptosis [9,53]. All of these agents act through modulating the immune system, although their mechanisms range from functional immune regulation and altered lymphocyte trafficking to selective sequestration or profound depletion of immune cell populations, which may have important implications for infection risk and virus–host interactions [9]. However, this review reveals that the therapeutic efficacy of DMTs in MS may extend beyond their established immunomodulatory and anti-inflammatory effects. Increasing evidence suggests that benefits may also be partially mediated through modulation of the interaction between EBV and the host immune system. From this perspective, MS therapies may act at two complementary levels: attenuating neuroinflammation and interfering with mechanisms sustaining viral latency and chronic immune activation. Accordingly, evaluation of therapeutic strategies should incorporate their impact on the EBV–B-cell–immune axis, increasingly recognized as a relevant component of MS pathophysiology. Emerging therapeutic strategies may further expand this perspective. In particular, Bruton’s tyrosine kinase (BTK) inhibitors, currently under extensive investigation in MS, appear especially interesting in the context of EBV-related mechanisms due to their effects on B-cell signaling, antigen presentation, microglial activation, and chronic compartmentalized inflammation within the CNS [54]. Importantly, BTK signaling is involved in multiple pathways associated with the activation and survival of B lymphocytes, including potentially EBV-infected memory B-cell populations considered crucial for MS pathogenesis [54,55]. Moreover, by targeting both peripheral immune responses and CNS-compartmentalized inflammation, BTK inhibitors may represent a therapeutic approach targeting mechanisms beyond typical immunomodulation [54,55]. Given the central role of EBV-infected B-cells in current pathogenetic models of MS [26], future studies should also evaluate the potential influence of BTK inhibitors on the EBV–host interaction and immune dysregulation

Across monoclonal antibody-based therapies, a consistent pattern is seen, indicating that clinical efficacy is not explained only by global immunosuppression but rather by selective modulation of EBV-related immune responses. Therapies targeting lymphocyte trafficking, such as integrin blockade, alter B-cell subset distribution, leading to peripheral accumulation of memory B-cells and antibody-secreting cells. This redistribution may increase the detectable humoral response against EBV without significantly affecting intrinsic functional properties. In contrast, anti-CD20 therapies exert a more direct influence on EBV-associated humoral immunity, reducing responses to latent viral antigens while largely preserving overall antiviral immunity. This supports the hypothesis that chronic antigen-driven stimulation mediated by EBV-infected B-cells is at least partially attenuated by B-cell depletion. Other immunomodulatory approaches, including those affecting cytokine signaling and T-cell trafficking, may primarily restore immune compartmental balance, reducing CNS-restricted EBV-specific effector responses and inflammatory mediators while redirecting immune activity toward the periphery. These observations suggest that therapeutic benefit in MS may partly arise from the disruption of EBV-driven immune persistence through complementary effects on cellular distribution, antigen-specific responses, and immune compartmentalization.

The therapeutic efficacy of both conventional and intensive MS treatments appears to be closely linked to their capacity to modulate the EBV–host dynamic through distinct yet partially converging cellular and molecular mechanisms. Among first-line therapies, IFN-β seems to primarily target the viral reservoir by reducing the memory B-cell load. Its overall impact appears largely confined to the cellular level, with stable anti-EBV antibody titers. Similarly, GA promotes a more balanced host–virus interaction by limiting the memory B-cell niche while reversing CD8+ T-cell exhaustion. In contrast, DMF appears to act predominantly by reducing the pathologically exaggerated immune response to EBNA-1, modulating T-cell reactivity and pro-inflammatory cytokine production without inducing broad immunosuppression.

In the context of intensive immune reconstitution, aHSCT does not rely on complete viral eradication but rather on a profound “immune reset,” leading to expansion and diversification of EBV-specific CD8+ T-cells. This re-established immune surveillance may more effectively control persistent viral antigens and potentially limit cross-reactive responses against CNS targets. Additional factors, such as vitamin D, may further modulate the EBV–immune interaction. Available data indicate that vitamin D can influence EBV-specific immune responses in MS; however, these effects appear modest and potentially transient. The long-term clinical relevance of this interaction remains uncertain and requires further investigation, particularly in relation to disease susceptibility and progression.

There are several concepts of MS targeting EBV. These include an EBNA1 inhibitor, which exerts a cytostatic effect on EBV-positive B lymphocytes, and MbCD, cholesterol inhibitors that limit EBV infection of astroglial cells and restrict the maintenance of viral latency. Furthermore, it is suggested that influencing proteolytic enzymes (Cathepsin G) and modulating MOG autophagy may reduce the activation of autoreactive T lymphocytes. Additionally, IgM monoclonal antibodies against MBP appear to produce an immunosuppressive effect and clinical improvement. Furthermore, emerging therapeutic approaches directly targeting EBV provide additional support for its role in MS pathogenesis. EBV-specific T-cell therapy has been associated with clinical improvements, including reductions in fatigue, decreased EDSS scores, and improved quality of life, without significant safety concerns. The proposed mechanism involves the elimination of EBV-infected B-cells within the CNS by administering CD8+ T-cells, resulting in reducing autoimmune activity and potentially promoting neurological recovery. Although these findings are promising, larger controlled studies are needed to confirm the efficacy and durability of this approach.

**Figure 2:**
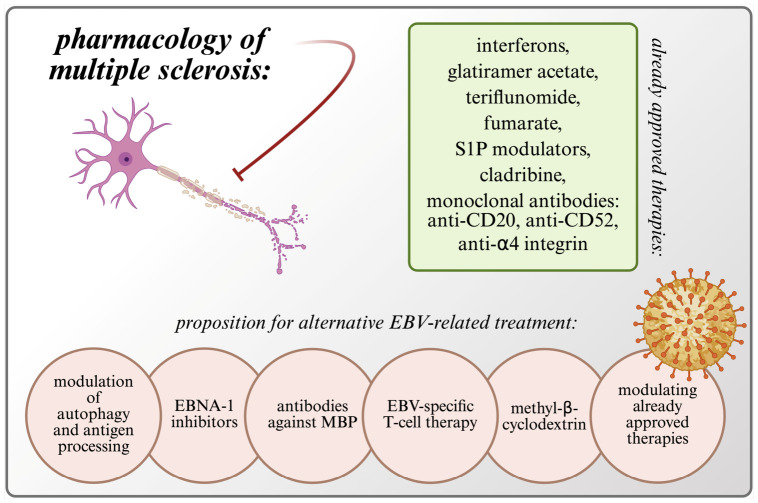
Simplified overview of the pharmacological landscape in multiple sclerosis and emerging EBV-related therapeutic approaches. EBV, Epstein–Barr virus; EBNA-1, Epstein–Barr nuclear antigen 1; MBP, myelin basic protein; S1P, sphingosine-1-phosphate. Figure created using BioRender.com under a licensed author account (O.G.).

Taken together, current DMTs in MS, while targeting the immune system through diverse mechanisms, may exert part of their therapeutic effect by modulating the EBV–host interaction, indicating that disruption of EBV-driven immune persistence represents a complementary pathway in MS treatment. This concept is further supported by emerging EBV-targeted therapies, such as EBV-specific T-cell approaches, which suggest that direct antiviral strategies may offer an additional therapeutic avenue (Figure 2).

Finally, EBV-related immune parameters have also been explored as potential predictors of treatment response in MS. Several studies suggest that changes in EBV-specific antibody titers and T-cell responses may correlate with therapeutic outcomes. Baseline immune profiles and longitudinal alterations in viral-specific immunity could potentially help identify patients more likely to benefit from specific treatments. However, findings remain inconsistent, and the predictive utility of these biomarkers is currently limited, warranting further validation in well-designed studies. Finally, currently available data do not definitively establish whether the observed changes in EBV-related markers result from direct effects of DMTs on EBV biology or rather represent secondary consequences of reduced inflammatory activity and immune system modulation associated with treatment efficacy in MS. More research is needed in order to potentially confirm the causality of observed relations.

## 5. Limitations

This review does not remain without some important limitations. First of all, the limited number of studies with significant heterogeneity between them limits robust and well-supported conclusions, highlighting the need for further well-designed studies. The differences in study design, population characteristics, interventions applied, and parameters measured lead to warranted caution while summarizing the findings. The number of patients in the included studies was often small, which should be taken into consideration beyond the significance of the results. Additionally, interpretation of the analyzed studies is limited by the inability to clearly distinguish causative EBV-specific therapeutic effects from indirect changes secondary to the reduction in inflammatory and immune processes induced by DMTs.

Regarding the applied methodology, there are also some limitations. The total number of four databases, although in general sufficient, does not use up all available resources. Despite the comprehensive database search and adherence to PRISMA-based methodology, the applied selection strategy and eligibility criteria may have led to the exclusion of some potentially relevant studies investigating EBV-related mechanisms of MS therapies, which could have influenced the overall comprehensiveness of the presented evidence. Furthermore, the decision to include only studies written in English, made in the view of potential readership, may lead to omitting important and relevant research published in other languages. Finally, there is a broad area of gray literature, which was not included in this review. Although this approach may increase the risk of publication bias, it was adopted to ensure a consistent level of methodological quality and data transparency.

## 6. Final Conclusions

DMTs in MS likely exert part of their clinical benefit through modulation of the EBV–B-cell–immune axis, not solely via nonspecific immunosuppression. Clinicians should recognize that different therapeutic classes may differentially influence EBV-related immune responses, which could have implications for the treatment selection and interpretation of immunological changes. Future research should focus on validating EBV-related biomarkers and clarifying whether direct targeting of EBV can provide sustained clinical benefit.

## Figures and Tables

**Table 1:** A summary of studies regarding the EBV-related mechanisms of monoclonal antibody function.

Ref.	Year	Population	Comparison	Therapy	EBV Involvement	Results in Treated Group
Clottu et al. [29]	2017	HC (n = 14); RRMS untreated pts (n = 14); RRMS NAT-treated pts (n = 11)	HC vs. RRMS; pre/post 9–12 m	NAT	EBI2 (EBV-induced receptor)	↑ EBI2 expression in memory CD4+ T-cells; ↑ migration to 7α,25-OHC; positive EBI2–migration correlation
Dungan et al. [30]	2024	RRMS pts (n = 86), epilepsy pts, HC	MS pts treated with NAT, anti-CD20, DMF vs. control groups	NAT, anti-CD20 (OCR/OMB), DMF	cellular response (IFN−γ and IL−2) to EBNA-1 peptides	↑ cellular responses in MS pts; ↓ cellular responses to EBNA-1 by anti-CD20 and DMF compared to control levels
Marti et al. [31]	2024	RRMS pts (n = 44): untreated pts (n = 19), NAT-treated pts (n = 10), aHSCT pts (n = 15); HC (n = 16)	MS pts vs. HC; pre-treatment vs. post-treatment	aHSCT and NAT	memory B-cell antibody response against EBNA1; cross-reactivity with GlialCAM and ANO2	NAT associated with ↑ peripheral memory B-cell responses; aHSCT leads to a long-term ↓ in humoral anti-EBNA1 reactivity
Rød et al. [32]	2023	RRMS pts (n = 99)	pre vs. 3–18 m follow-up	OCR/RTX	latent EBV in memory B-cells; EBNA-1, VCA-p18, EBV-DNA	↓ EBNA-1 IgG; ↑ VCA IgG; slight ↓ CMV IgG/total IgG; rare EBV-DNA+
Pham et al. [33]	2023	MS pts (n = 36; RRMS/SPMS/PPMS)	pre vs. 12 m post	OCR	latent EBV antibody response in B-cells	↓ EBNA-1 Ab; ↓ BFRF3 Ab; gp350 and total IgG unchanged
Wuest et al. [34]	2014	RRMS pts (n = 40); PMS pts (n = 41); untreated OIND pts (n = 19)	untreated vs. DAC HYP pts; CSF vs. blood	DAC HYP	intrathecal EBV-specific CD4+/CD8+ T-cell enrichment	normalized CSF/blood EBV response; ↓ intrathecal dominance
Jagessar et al. [35]	2013	marmoset twins with EAE (n = 5)	no control	anti-CD20, anti-BLyS, anti-APRIL	CalHV3-infected B-cells	↓ of infected B-cells after anti-CD20. no effect of anti-BLyS and anti-APRIL

↑, increased; ↓, decreased; Ab, antibodies; aHSCT, autologous Hematopoietic Stem Cell Transplant; ANO2, anoctamin 2; APRIL, A Proliferation-Inducing Ligand; autoAg, autoantigen; BFRF3, BamHI F rightward reading frame 3; BLyS, B-Lymphocyte Stimulator; CalHV3, Callithrix herpesvirus 3; CMV, cytomegalovirus; CSF, cerebrospinal fluid; DAC HYP, daclizumab high-yield process; DMF, dimethyl fumarate; EAE, experimental autoimmune encephalomyelitis; EBNA-1, Epstein–Barr nuclear antigen 1; EBV, Epstein–Barr virus; EBI2, Epstein–Barr virus-induced gene 2; GlialCAM, glial cell adhesion molecule; HC, healthy controls; IFN-γ, interferon gamma; Ig, immunoglobulin; IL, interleukin; m, months; MS, multiple sclerosis; NAT, natalizumab; OCR, ocrelizumab; OIND, other inflammatory neurological diseases; PMS, progressive multiple sclerosis; PPMS, primary progressive multiple sclerosis; pts, patients; Ref., reference; RRMS, relapsing–remitting multiple sclerosis; RTX, rituximab; SPMS, secondary progressive multiple sclerosis; VCA, viral capsid antigen; vs, versus.

**Table 2:** A summary of studies regarding the EBV-related mechanisms of function of therapies used in multiple sclerosis other than monoclonal antibodies.

Ref.	Year	Population	Comparison	Therapy	EBV Involvement	Effects
Rizzo et al. [36]	2016	RRMS pts (n = 35)	baseline vs. 1 and 6 m post-therapy	IFN-β	LMP2A gene expression in PBMCs	↓ pathogenic memory B-cells via FAS-mediated apoptosis and ↓ LMP2A gene expression
Lande et al. [37]	2008	RRMS pts (n = 9), SPMS pts (n = 2), post-mortem brain cases (n = 4)	baseline vs. 1 day and 90 days post-therapy	IFN-β	pDCs and MxA expression	impaired pDC maturation and their response to stimuli by IFN-β; ↑ of inhibitory B7H1 by IFN-β; results suggesting EBV-infected B-cells in the brain act as a maturation stimulus for pDCs
Lie et al. [39]	2023	RRMS pts (n = 84)	baseline vs. 18 m post-initiation	IFNβ-1a	serum antibodies (EBNA-1 IgG, EA IgG, VCA IgG, VCA IgM)	NS associations between treatment and serum anti-EBV antibody levels
Guerrera et al. [40]	2020	RRMS pts (n = 77) (35 longitudinal); HC (n = 48)	GA-treated vs. untreated pts vs. HC	GA	EBV-specific CD8 T lymphocytes and B-cells	recognition by GA by ↑ virus-specific CD8 T-cells and ↓ their exhaustion; memory B-cell frequency ↓ by GA
Massey et al. [41]	2023	MS (n = 13)	pre-aHSCT pts vs. 6, 12, and 24 m post-aHSCT pts	aHSCT	viremia, B-cell EBV genome load, CTL responses (TCR repertoire)	↑ and diversification of EBV-specific CD8 CTL responses; ↑ B-cell EBV load not associated with relapse
Najafipoor et al. [42]	2015	vitamin D3-treated RRMS pts (n = 27)	RRMS (n = 13)	50,000 IU of vitamin D3 per week for 6 m	anti-EBNA-1, anti-VCA	lower ↑ IgG levels against EBNA1 and VCA compared to the control
Røsjø et al. [43]	2016	vitamin D3-treated RRMS pts (n = 35)	RRMS (n = 33)	20,000 IU of vitamin D3 per week for 96 weeks	anti-EBNA-1	↓ anti-EBNA1 from baseline to 48. week vs. control; NS differences from baseline to week 96

↑, increased; ↓, decreased; aHSCT, autologous Hematopoietic Stem Cell Transplant; CTL, cytotoxic T lymphocytes; EA, early antigen; EBNA-1, Epstein–Barr nuclear antigen 1; EBV, Epstein–Barr Virus; GA, glatiramer acetate; HC, healthy controls; IFN, interferon; Ig, Immunoglobulin; IU, International Unit; LMP2A, latent membrane protein 2A; m, mothts; MS, multiple sclerosis; MxA, Myxovirus resistance protein A; NS, no statistical; PBMC, peripheral blood mononuclear cells; pDCs, plasmacytoid dendritic cells; pts, patients; Ref, reference; RRMS, relapsing–remitting multiple sclerosis; SPMS, secondary progressive multiple sclerosis; TCR, T-cell receptors; VCA, viral capsid antigen; vs, versus.

**Table 3:** A summary of studies regarding EBV-based therapies.

Ref.	Year	Population	Comparison	Therapy	EBV Involvement	Results in Treated Group
Ioannides et al. [44]	2021	PPMS pts (n = 5); SPMS pts (n = 3)	no control group	autologous EBV-specific T-cell therapy	elimination of EBV-infected B-cells	↓ fatigue, ↓ EDSS score
Pender et al. [45]	2018	PPMS pts (n = 8); SPMS pts (n = 5); disease duration of 11.8 ± 7.7 years	no control group	EBV-specific T-cell therapy (EBNA1, LMP1, LMP2A)	depletion of EBV-infected B-cells in the CNS	↓ fatigue, improved quality of life, ↓ EDSS score, ↓ IgG in the CSF
Monaco et al. [46]	2023	sMS pts (n = 25); aMS pts (n = 25); HC (n = 26)	SMS vs. aMS; MS vs. HC; EBNA1 inhibitor vs. CdA	EBNA-1 inhibitor; CdA	EBNA-1-mediated persistence of EBV in B-cells	EBV in B-cells: aMS- 80%;(20/25); sMS -28%;(7/25); HC- 42%;(11/26); EBNA-1 inhibitor: ↓ EBNA-1 binding to EBV DNA, cytostatic effect, ↓ metabolic activity only in EBV+ cells; non-selective effect of CdA
Morandi et al. [47]	2017	HC (n = 8)	EBV (+) vs. EBV (−) lymphocytes B	modulation of autophagy and proteolytic enzymes	effect of EBV on: HLA-I, HLA-II, CD80, CD86, MOG CatG, and autophagy	↑ HLA-I, HLA-II, CD80, CD86; autophagy and CatG activation, MOG degradation
Rani et al. [48]	2023	in vitro cell line models (LN-229, HEK 293T)	EBV (+) vs. EBV (−) LN-229 astroglial cells	MbCD	EBV entry into cells via membrane cholesterol	↓ EBV-GFP, EBNA1, LMP1, LMP2A; ↓ STAT3, RIP, NF-κB and TNF-α
Annunziata et al. [49]	2013	RRMS pts (n = 47); SPMS pts (n = 13); OND pts (n = 22); HC (n = 20)	MS vs. OND vs. HC	monoclonal IgM anti-MBP (105–120) targeting CD64	IgM produced by EBV (+) B-cells	clinical improvement in pts with anti-MBP IgM antibodies, ↓ T-cell proliferation; ↑ IL-10; ↓ IL-12

↑, increased; ↓, decreased; aMS, active multiple sclerosis; anti-MBP, anti-myelin basic protein; CatG, Cathepsin G; CdA, cladribine; CD80/CD86, costimulatory molecules; CNS, central nervous system; CSF, cerebrospinal fluid; EBNA-1, Epstein–Barr nuclear antigen 1; EBV-GFP, Epstein–Barr Virus expressing Green Fluorescent Protein; EDSS, Expanded Disability Status Scale; HC, healthy controls; HLA-I/HLA-II, Human Leukocyte Antigen class I/II; IgM, Immunoglobulin M; IL, interleukin; LMP1/LMP2A, Latent Membrane Protein 1/2A; MbCD, methyl-β-cyclodextrin; MOG, myelin oligodendrocyte glycoprotein; NF-κB, nuclear factor kappa B; OND, Other Neurological Disease; PPMS, primary progressive multiple sclerosis; pts, patients; Ref., reference; RIP, receptor-interacting protein; RRMS, relapsing–remitting multiple sclerosis; sMS, stable multiple sclerosis; SPMS, secondary progressive multiple sclerosis; STAT3, signal transducer and activator of transcription 3; TNF-α, Tumor Necrosis Factor alpha; vs, versus.

**Table 4:** A summary of studies regarding EBV-related patterns of treatment effectiveness.

Ref.	Year	Population	Comparison	Therapy	EBV Involvement	Effects
Comabella et al. [50]	2012	MS pts (n = 28)	no control	IFN-β for 1 year	humoral and cellular immune responses to EBV-encoded antigens	NS ↓ of anti-EBNA1, no change in anti-VCA. ↓ of EBNA1-specific CD4+ T-cell responses, no change in CD8+ T-cell
Dominguez-Mozo et al. [51]	2023	RRMS pts (n = 101), from which 13 stopped and 8 had less than 24 months of follow-up	no control	TER for 24 months	anti-EBNA-1, anti-VCA	↓ of anti-EBNA-1 and VCA NS association between antibody variation and the clinical and radiological responses older starting age of the treatment and ↑ EBNA-1 titers as early predictors of NEDA-3 in TER-treated MS pts
Dominguez-Mozo et al. [52]	2020	MS pts (n = 186)	no control	NAT for 2 years	anti-EBNA-1, anti-VCA	predictors of early treatment response: a baseline EDSS score below 3.0, ↓ initial levels of anti-EBV antibodies, and ↓ HHV-6 IgG titers

↑, increased; ↓, decreased; EBNA-1, Epstein–Barr nuclear antigen 1; EBV, Epstein–Barr Virus; EDSS, Expanded Disability Status Scale; HHV-6, Human Herpesvirus 6; IFN-β, interferon beta; IgG, Immunoglobulin G; MS, multiple sclerosis; NAT, natalizumab; NEDA-3, No Evidence of Disease Activity-3; NS, no significant; pts, patients; Ref., reference; RRMS, relapsing–remitting multiple sclerosis; TER, teriflunomide; VCA, viral capsid antigen.

## Data Availability

All data supporting the findings of this study are included in the article and its Supplementary Materials.

## Supplementary Materials

The following supporting information can be downloaded at: https://www.mdpi.com/article/10.3390/jcm15114104/s1, Table S1. PRISMA checklist [56]. Table S2. Risk of bias assessment.

## Abbreviations

The following abbreviations are used in this manuscript:

MS	multiple sclerosis
CNS	central nervous system
DMTs	Disease-modifying therapies
RRMS	Relapsing–remitting multiple sclerosis
EBV	Epstein–Barr virus
Ig	immunoglobulin
VCA	viral capsid antigens
EBNA	EBV nuclear antigens
RNA	ribonucleic acid
MHC	major histocompatibility complex
HLA	human leukocyte antigen
gp	glycoprotein
PRISMA	Preferred Reported Items for Systematic Reviews and Meta-Analyses
EDSS	Expanded Disability Status Scale
MRI	magnetic resonance imaging
CSF	cerebrospinal fluid
PBMCs	peripheral blood mononuclear cells
IL	interleukin
GlialCAM	glial cell adhesion molecule
CMV	cytomegalovirus
DNA	deoxyribonucleic acid
DAC HYP	daclizumab high-yield process
IFN	interferon
TNF	tumour necrosis factor
pDCs	plasmacytoid dendritic cells
EA	early antigen
GA	glatiramer acetate
DMF	dimethyl fumarate
BLyS	B-lymphocyte stimulator
APRIL	a proliferation-inducing ligand
aHSCT	autologous hematopoietic stem cell transplantation
LMP	latent membrane protein
MOG	myelin oligodendrocyte glycoprotein
MbCD	methyl-β-cyclodextrin
MBP	myelin basic protein
HHV-6	human herpes virus 6
