# Supplementary material for: Experimental Therapies in Multiple Sclerosis: Epstein–Barr Virus and Potential EBV-Related Therapeutic Strategies—A Systematic Review
Source: J Clin Med. 2026 May 26;15(11):4104. doi: 10.3390/jcm15114104 (PMC13258446; doi:10.3390/jcm15114104)
Supplement: Supplementary file 1 [file jcm-15-04104-s001.zip › jcm-4326610-supplementary/Table S2. Risk of bias assessment.pdf]

Supplementary Table S2. The assessment of methodological quality and risk of bias of the included studies using the **Newcastle-Ottawa Scale.** The evaluation was based on three domains: selection, comparability and outcome. Scores of 7 to 9 points mean low risk of bias, 4 to 6 points moderate risk of bias, and 0 to 3 points high risk of bias.

Study	Selection (0-4)	Comparability (0-2)	Outcome (0-3)	Total (0-9)
**Clottu et al. [29]**	★★★★	★	★★	7/9
**Dungan et al. [30]**	★★★	★	★★	6/9
**Marti et al. [31]**	★★★	★	★★	6/9
**Rød et al. [32]**	★★★★	★	★★★	8/9
**Pham et al. [33]**	★★★	★	★★★	7/9
**Wuest et al. [34]**	★★★★	★	★★★	8/9
**Jagessar et al. [35]**	★★★	★	★★	6/9
**Rizzo et al. [36]**	★★★	★	★★	6/9
**Lande et al. [37]**	★★	★	★★	5/9
**Lie et al. [39]**	★★★★	★★	★★	8/9
**Guerrera et al. [40]**	★★★	★★	★★	7/9
**Massey et al. [41]**	★★	★	★★	5/9
**Najafipoor et al. [42]**	★★★	★	★★	6/9
**Røsjø et al. [43]**	★★★★	★★	★★★	9/9
**Ioannides et al. [44]**	★★★	0	★★	5/9
**Pender et al. [45]**	★★★	0	★★	5/9
**Monaco et al. [46]**	★★★★	★	★★	7/9
**Morandi et al. [47]**	★★★	0	★★	5/9
**Rani et al. [48]**	★★	0	★★	5/9
**Annunziata et al. [49]**	★★★	★	★★	6/9
**Comabella et al. [50]**	★★	0	★★	4/9
**Dominguez-Mozo et al. [51]**	★★★	★	★★★	7/9
**Dominguez-Mozo et al. [52]**	★★★	★	★★★	7/9
